# Intra-articular venous malformation of the knee in children: magnetic resonance imaging findings and significance of synovial involvement

**DOI:** 10.1007/s00247-019-04580-5

**Published:** 2019-12-19

**Authors:** Katariina A. Mattila, Johanna Aronniemi, Päivi Salminen, Risto J. Rintala, Kristiina Kyrklund

**Affiliations:** 1 Department of Pediatric Surgery, Children’s Hospital, Helsinki University Hospital and University of Helsinki, P.O. Box 347, 00029 HUS, Helsinki, Finland; 2 Department of Radiology, HUS Medical Imaging Center, Helsinki University Hospital and University of Helsinki, Helsinki, Finland; 3 VASCERN VASCA European Reference Center, Helsinki University Hospital, Helsinki, Finland

**Keywords:** Children, Intra-articular, Knee, Magnetic resonance imaging, Synovium, Venous malformation

## Abstract

**Background:**

Intra-articular venous malformations of the knee are an uncommon cause of unilateral knee pain in children. Timely diagnosis is important because lesions with intrasynovial involvement can lead to joint space hemorrhage and secondary cartilage damage.

**Objective:**

To describe our tertiary center’s experience of diagnostics and typical magnetic resonance imaging (MRI) findings.

**Materials and methods:**

A retrospective review of all patients ≤16 years of age managed for intra-articular venous malformations of the knee at our institution between 2002 and 2018.

**Results:**

Of 14 patients (8 male), the mean age at presentation was 6 years (range: 0–14 years). The most common clinical findings were unilateral knee pain (93%), joint swelling (79%), quadriceps atrophy (50%) and a limited range of motion (29%). Cutaneous manifestations were present in four patients (29%). Contrast-enhanced MRI was available in all cases. After initial MRI, a vascular anomaly etiology had been identified in 11 cases (79%), and correctly reported as a venous malformation in 6 (55%). Three patients received entirely different diagnoses (arthritis, tumor or pigmented villonodular synovitis). Three of seven patients with intrasynovial lesions had established chondropathy at diagnosis. Two patients with lesions of the suprapatellar fat pad had intrasynovial involvement that was not visualised on MRI.

**Conclusion:**

Although MRI usually permits the diagnosis, clinical awareness of these lesions is important for optimal imaging, accurate interpretation and timely diagnosis. Involvement of the intrasynovial cavity carries a risk of hemarthrosis and progressive chondropathy that may be underestimated by MRI.

## Introduction

**Fig. 1:**
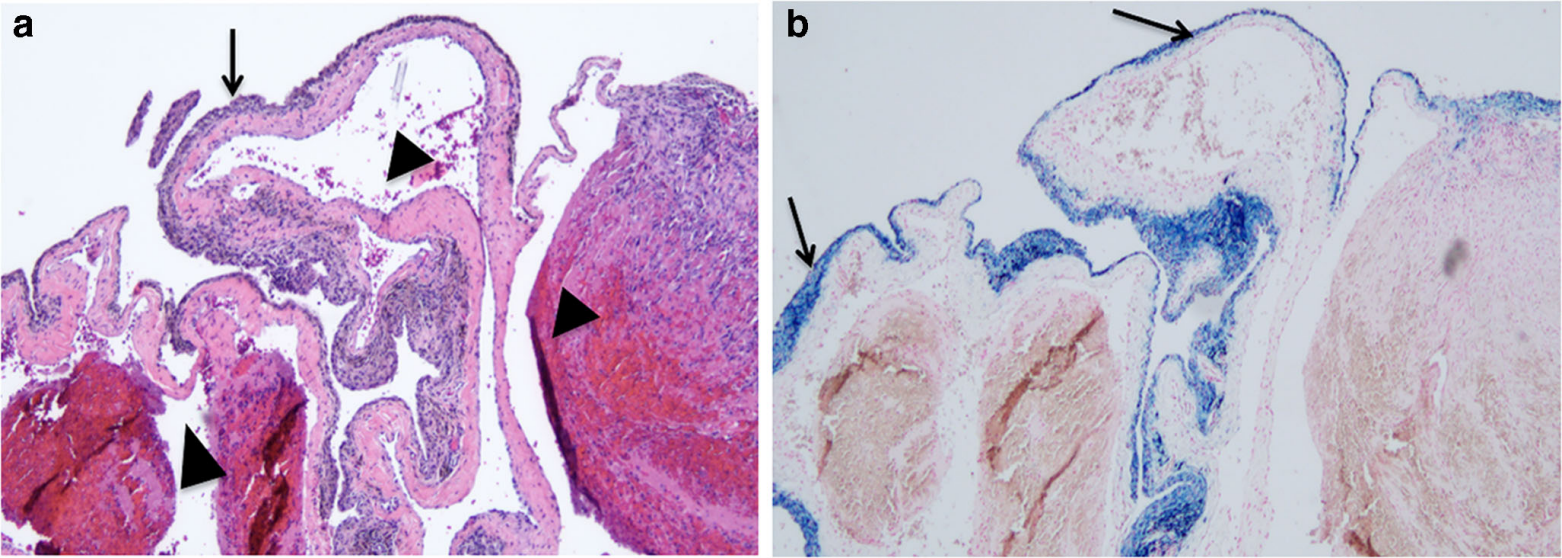
Paraffin sections of a resected synovial venous malformation demonstrate post-hemorrhage iron accumulation at the synovium. **a** The synovium is staining dark purple at herovici staining (*arrow*). Dilated venous spaces with intraluminal thrombi are right beneath the synovium (*arrowheads*). **b** At Prussian blue staining, synovial iron stains blue (*arrows*). Fig. 1a and b are a 40x magnification. Image courtesy of Dr. Jouko Lohi

Venous malformations are benign congenital, slow-flow vascular anomalies consisting of thin-walled and dilated venous channels [1, 2]. Intra-articular venous malformations of the knee are a rare subtype of venous malformations that are an uncommon cause of knee pain in children. Presenting symptoms often include nonspecific pain and joint swelling, which may be episodic, accompanied by a limited range of motion. Secondary muscle atrophy may be present if symptoms are prolonged. External signs suggestive of a venous malformation may include varicosities or capillary malformations on the skin surface or, more rarely, overgrowth or undergrowth of the affected limb. However, superficial skin changes may also be absent, and the symptoms and signs display overlap with those of other more common conditions including juvenile idiopathic arthritis, trauma or even tumors [3, 4]. Timely diagnosis of intra-articular venous malformations of the knee is essential because lesions with intrasynovial involvement can lead to joint space hemorrhage and secondary cartilage damage, which is the most significant complication [4–7] (Fig. 1). Hence, improving clinical awareness of venous malformations and familiarity with their magnetic resonance imaging (MRI) characteristics are beneficial.

This study aims to highlight intra-articular venous malformation of the knee as a rare but important differential diagnostic cause of unilateral knee pain in children. We describe our institutional experience in diagnosing these lesions, as well as their typical MRI findings against other diagnoses. We also emphasize the importance of evaluating and recognizing possible synovial involvement.

## Materials and methods

This study was carried out at the Children’s Hospital, Helsinki University Hospital, which is a tertiary referral centre for vascular anomalies in children. Helsinki University Hospital founded an interdisciplinary team in 2002 to diagnose and treat vascular anomalies. The team includes diagnostic and interventional radiology, pediatric, plastic, maxillofacial and orthopedic surgery, oncology, otorhinolaryngology, ophthalmology, dermatology, genetics and pathology.

The case records and imaging of all children ≤16 years of age with intra-articular venous malformations of the knee managed at our institution between 2002 and 2018 were retrospectively reviewed. Referral diagnoses were compared with definitive diagnoses established by our vascular anomaly team. Definitive diagnoses were based on the updated International Society for the Study of Vascular Anomalies (ISSVA) classification [8]. MRI studies were analyzed for morphology, enhancement and anatomical locations of the lesions, and to review the need for further imaging for diagnostics. Lesions with entire or partial location within the joint capsule were considered intra-articular. Based on imaging, intra-articular lesions were further subdivided into: 1) intracapsular but extrasynovial, including the suprapatellar and infrapatellar fat pads, or 2) intracapsular and intrasynovial, when entirely or partially affecting the intrasynovial cavity. Involvement of extracapsular tissues was also recorded.

## Results

### Clinical data

Fourteen pediatric patients (8 males) with intra-articular venous malformation of the knee were identified. The mean age at presentation was 6 years (range: 0–14 years). The most common presenting symptoms and signs were unilateral knee pain (13/14), joint swelling (11/14), quadriceps atrophy (7/14) and a limited range of motion (4/14). Cutaneous manifestations suggestive of a vascular anomaly were present in four patients: superficial varicosities in one, and regional skin surface capillary malformations in three. The D-dimer level, which may be elevated in venous malformations due to localized intravascular coagulopathy [7, 9], was raised (> 0.5 mg/l) in nine patients (mean: 1.7 mg/l, range: 0.0–5.5).

### Referral diagnoses and initial magnetic resonance imaging diagnoses

The mean time from the onset of symptoms to the definitive diagnosis was 2 years (range: 0–9 years). Patient characteristics, referral diagnoses and initial diagnoses following MRI are presented in Table 1. After initial MRI, a vascular anomaly etiology was identified in 11 patients (79%), and correctly reported as a venous malformation in 6 patients (55%), an unspecified vascular anomaly in 2 patients (18%) and incorrectly as a hemangioma in 3 patients (27%). Three patients received entirely other diagnoses based on initial clinical assessment and imaging (arthritis, tumor or pigmented villonodular synovitis) and were referred after a delay following tissue biopsy or assessment of joint aspirate. The MRI protocol varied depending on the imaging practices of the referral units. In cases with insufficient sequences for tissue characterization (T1- and T2-weighted turbo spin echo and contrast-enhanced sequences), the study was subsequently performed at our institution.

**Table 1:** Patient characteristics and time to diagnosis

Patient	Age at first contact	Time to diagnosis	Referral diagnosis	First magnetic resonance imaging report	Diagnosis after assessment by vascular anomalies team and/or further imaging
1	2 years	5 months	arthritis	venous malformation	venous malformation
2	7 years	2 months	reactive arthritis	hemangioma	venous malformation
3	2 years	2 years, 4 months	juvenile idiopathic arthritis	pigmented villonodular synovitis	venous malformation
4	4 years	4 years, 7 months	juvenile idiopathic arthritis	arthritis	venous malformation
5	8 years	3 years, 10 months	juvenile idiopathic arthritis	venous malformation	venous malformation
6	10 years	0 months	hemangioma	hemangioma	venous malformation
7	7 years	9 years, 1 month	hemangioma	hemangioma	capillary-venous malformation
8	4 years	2 months	tumour	tumour	venous malformation
9	0 years	0 months	tumour	unspecified vascular anomaly	capillary-venous malformation
10	3 years	0 months	unspecified vascular anomaly	unspecified vascular anomaly	venous malformation
11	6 years	2 years, 8 months	venous malformation	venous malformation	venous malformation
12	14 years	0 months	venous malformation	venous malformation	venous malformation
13	12 years	0 months	venous malformation	venous malformation	venous malformation
14	11 years	0 months	capillary-venous malformation	venous malformation	capillary-venous malformation

### Imaging findings

**Fig. 2:**
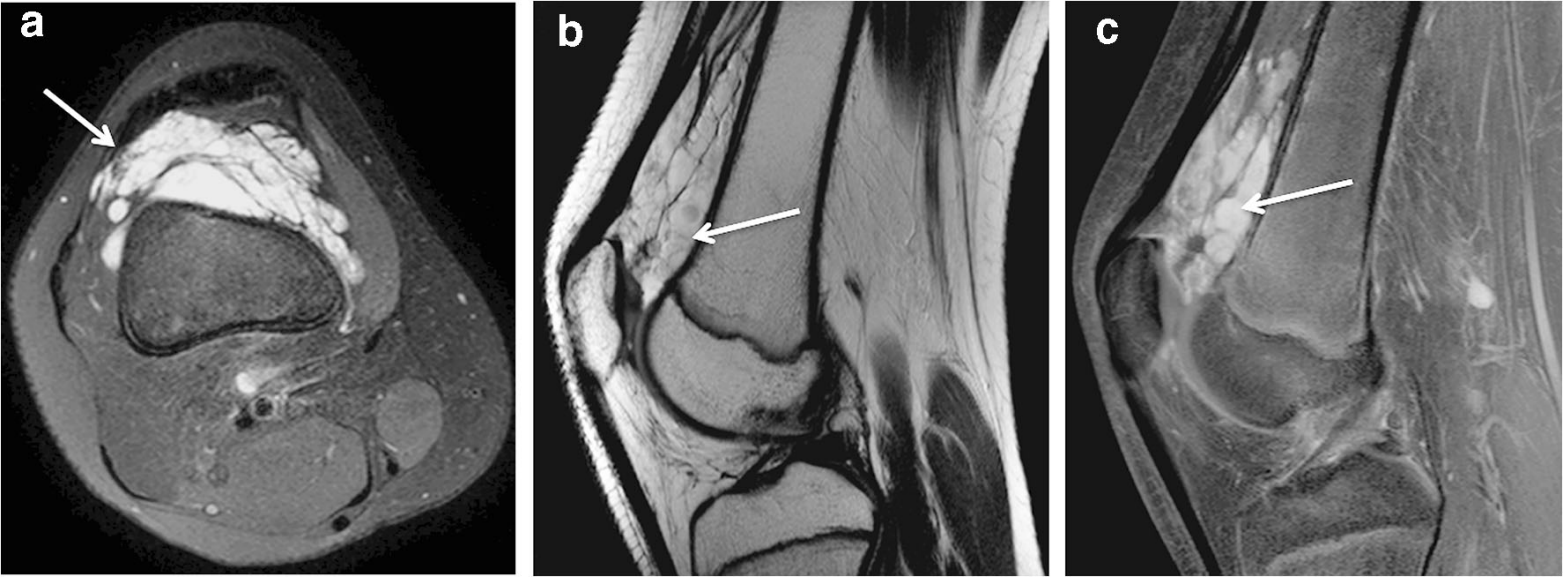
A 12-year-old girl with a venous malformation of the suprapatellar fat pad. **a** A T2-weighted fat-saturated axial MRI shows a typical cluster-like lesion consisting of tubular structures (*arrow*). **b** A T2-weighted sagittal MRI demonstrates the close proximity of the suprapatellar synovial recess and the venous malformation. The outline of the synovium is not identifiable between the venous malformation and femoral cortex (*arrow*) and possible synovial involvement cannot be ruled out. **c** A T1-weighted fat-saturated MRI shows partial enhancement of the lesion after gadolinium injection (*arrow*)

**Fig. 3:**
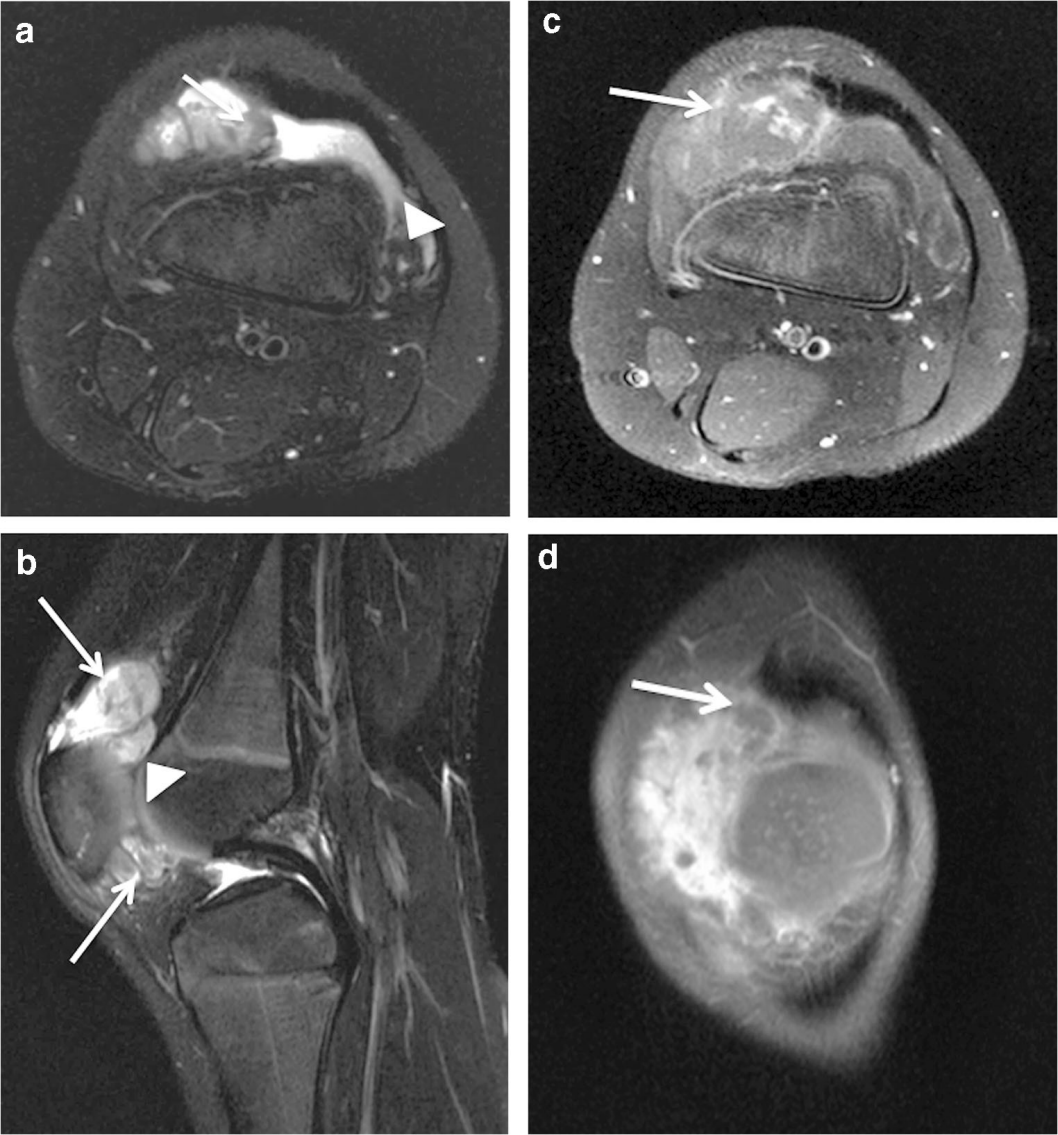
A 7-year-old boy with an intraarticular and intrasynovial venous malformation of the knee. **a**, **b** Axial (**a**) and sagittal (**b**) T2-weighted fat-saturated MR images. The cluster-like venous malformation protrudes into the suprapatellar recess (*arrow* in **a**). A mild synovial effusion with post-hemorrhage debris is present (*arrowhead* in **a**). The venous malformation invades both the suprapatellar and infrapatellar fat pads (*arrows* in **b**) as well as the intrasynovial cavity (*arrowhead* in **b**). **c**, **d** T1-weighted fat-saturated post-contrast axial (**c**) and coronal (**d**) MR images demonstrate the progressive enhancement typical for venous malformations. The first post-contrast image (**c**) shows only mild and partial enhancement of the venous malformation (*arrow*). A consecutively obtained coronal image (**d**) demonstrates more extensive enhancement. The filling defects (*arrow* in **d**) are due to intraluminal thrombosis often present in venous malformations

**Fig. 4:**
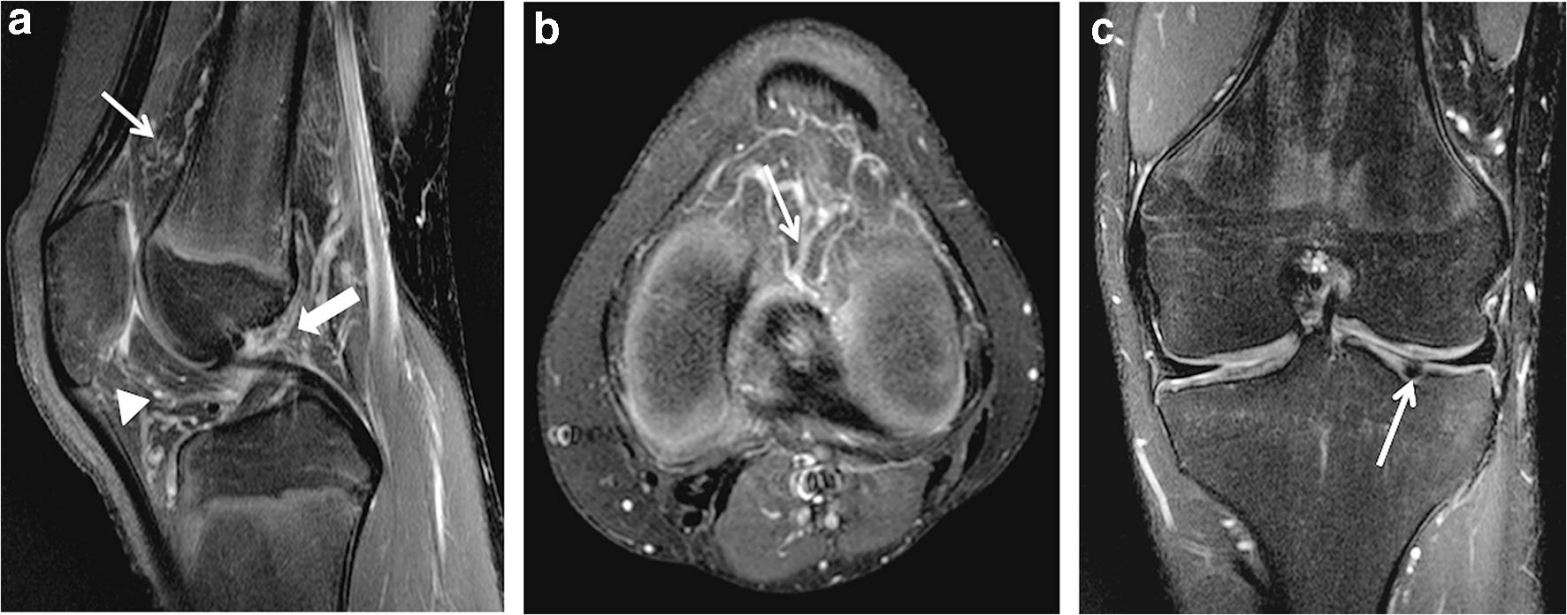
A 13-year-old boy with a diffuse intra-articular venous malformation of the knee. **a** A T2-weighted fat-saturated sagittal MRI shows the diffusely infiltrating phlebectatic vessels affecting the suprapatellar fat pad (*thin arrow*), infrapatellar fat pad (*arrowhead*) and intrasynovial cavity at the level of intercondylar space (*thick arrow*). **b** An axial T2-weighted fat-saturated MRI at the level of the intercondylar space demonstrates the pathological veins invading the intercondylar space and thus intrasynovial cavity (*arrow*). **c** A T2-weighted fat-saturated coronal MRI demonstrates thinning of cartilages and a full-thickness cartilage defect (*arrow*) of the same knee developed as a result of recurrent intrasynovial hemorrhages

**Fig. 5:**
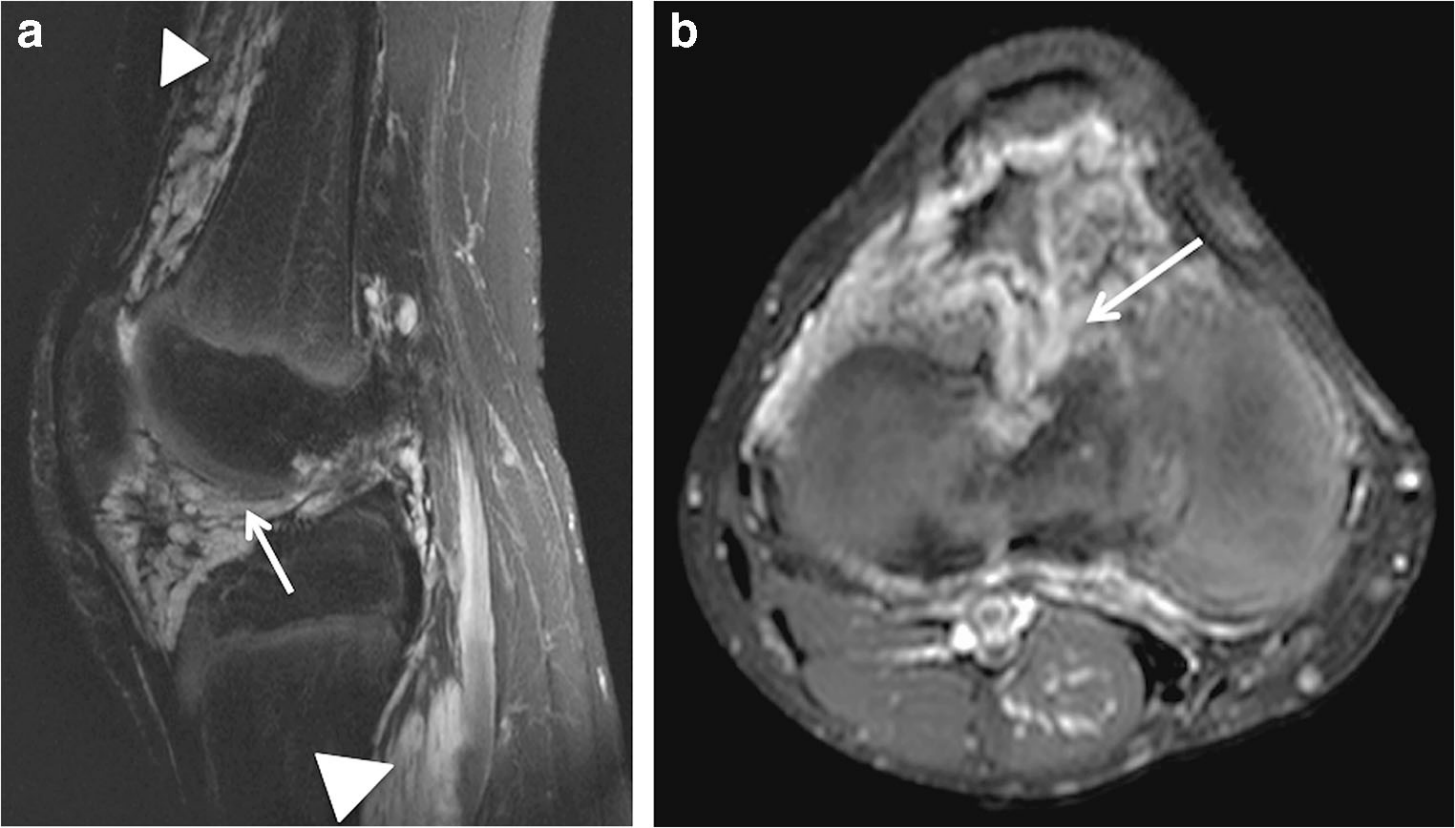
An 11-year-old boy with an extensive and diffuse venous malformation of the lower extremity. **a** A T2-weighted fat-saturated sagittal MRI demonstrates the involvement of all intracapsular spaces including the synovial cavity in the intercondylar space (*arrow*), as well as extracapsular muscle involvement (*arrowheads*). **b** A T1-weighted fat-saturated post-contrast axial MRI at the level of the intercondylar space demonstrates intensive enhancement of the ectatic veins of the malformation (*arrow*)

**Fig. 6:**
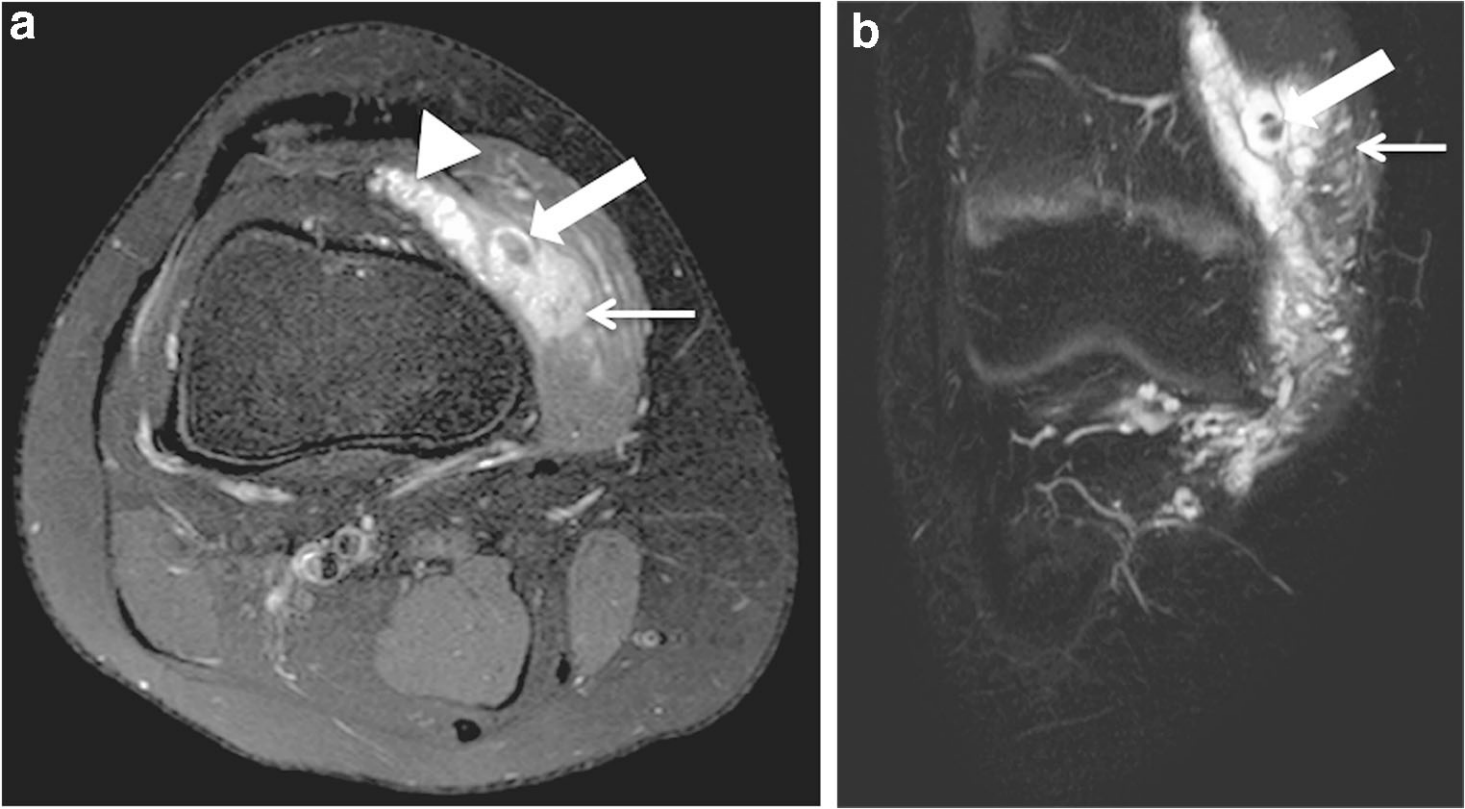
A 13-year-old girl with a venous malformation of the knee that affects both intra- and extracapsular tissues. **a**, **b** T2-weighted fat-saturated axial (**a**) and coronal (**b**) magnetic resonance images demonstrate the venous malformation invading the distal end of the vastus medialis muscle (*thin arrows*) and the suprapatellar fat pad (*arrowhead*). The small low-signal foci are consistent with intravascular thrombi or phleboliths (*thick arrows*)

Contrast-enhanced MRI (1.5 T) of the knee was assessed in all patients. The venous malformations appeared as collections of tubular structures with high signal intensity on T2-weighted sequences (Fig. 2). The lesion morphology was well demarcated and cluster-like in half of the patients (Figs. 2 and 3), the other half being diffuse and phlebectatic (Figs. 4 and 5). The enhancement pattern after gadolinium was partial and progressively increased in consecutive sequences in 10 patients (Fig. 3). However, in diffuse lesions with narrower vascular spaces, the enhancement was intense beginning from the first post-contrast images (Fig. 5). Phleboliths or thrombosis was present in 79% of the lesions (Figs. 3 and 6).

The suprapatellar fat pad was the most common location affected (12/14 patients) (86%) (Fig. 2). Overall, the venous malformation appeared intracapsular but extrasynovial in 7 patients (50%) and intracapsular with intrasynovial extension in the remaining 7 (Figs. 2, 3, 4 and 5). A synovial effusion was present in 4 patients (29%), all with intrasynovial involvement (Fig. 3). Three of the 7 clearly intrasynovial lesions (43%) were associated with established chondral damage, characterized by thinning of the articular surface cartilage, full thickness cartilage defects and osteochondral lesions (Fig. 4). Extracapsular tissues (muscle, subcutaneous fat, bone) were affected in 10 of the patients (71%; Figs. 5 and 6). Among the 12 patients with suprapatellar fat pad lesions, 2 lesions that appeared to have only extrasynovial components on MRI were found to have intrasynovial extension during subsequent operative management. In these cases, no synovial effusion was present at the time of MRI (Fig. 2).

## Discussion

Intra-articular venous malformation of the knee is a rare but important entity in children. Consistent with the literature and our series, knee pain and swelling due to intra-articular venous malformations may be mistaken for juvenile idiopathic arthritis [3, 4], pigmented villonodular synovitis or tumors. Cutaneous changes, including superficial venous or capillary malformations, were important clinical clues that led to identifying the venous malformation or at least suspicion of a vascular anomaly as the etiology; however, external signs were only present in 29% of cases. In pediatric patients, persistent unilateral knee pain is an abnormal symptom in any case that warrants further investigation. Clinical awareness of intra-articular venous malformations among the spectrum of unusual diagnoses is perhaps most important in prompting further imaging. MRI is pivotal in the diagnostics of intra-articular venous malformation of the knee [10] and in evaluating their anatomical location and possible sequelae. In the context of venous malformations of the limb, clinical risk factors for subsequent joint dysfunction were an age younger than 10 years, and pain duration over 1 year according to a recent series [11].

The imaging work-up of nonspecific knee pain in pediatric patients should always include a plain film x-ray, which is often normal in intra-articular venous malformations. Phleboliths, when present, may be visible on x-ray and raise the suspicion of a venous malformation. The MRI protocol for vascular anomalies should include T1 and T2 turbo spin echo sequences with and without fat saturation, and T1 fat-saturated post-contrast sequences to enable tissue characterization. Our MRI protocol for nontraumatic knee conditions is appropriate for venous malformation of the knee as well. The protocol includes: T2 fat-saturated axial, proton density coronal, T1 turbo spin echo coronal, T2 turbo spin echo sagittal, and T1 fat-saturated pre- and post-contrast images.

On MRI, venous malformations typically appear as tubular structures that display high signal intensity in T2-weighted and intermediate to low signal intensity in T1-weighted sequences (Fig. 2). The morphology of venous malformations is best evaluated in T2-weighted fat-saturated sequences and varies from lobulated cluster-like structures (Fig. 2) to diffusely infiltrating phlebectasia (Figs. 4 and 5) [12, 13]. Phleboliths, the small intravenous calcifications, appear as small signal void foci in both T1- and T2-weighted sequences (Fig. 6). They are hallmarks for venous malformations but are not uniformly present [14]. T2 turbo spin echo sequence in the sagittal plane is particularly useful in evaluating the exact anatomy of the malformation and possible synovial involvement in the intercondylar space or suprapatellar recess (Fig. 2). Intravenous gadolinium helps characterize tissue and confirm the vascular nature of the lesion; as venous malformations are connected to the systemic circulation via capillaries, they fill with the contrast agent. The filling rate is variable depending on the flow velocity and the size of the vascular spaces. Delayed (6–7 min) post-contrast images are helpful because the enhancement typically increases gradually in consecutive sequences following injection (Fig. 3). Filling defects due to thrombosis may be present (Fig. 3), whereas organizing thrombi tend to enhance intensively. In suspicion of high flow vascular lesions, arteriovenous malformations and fistulas, a dynamic contrast-enhanced magnetic resonance angiography is essential to detect arteriovenous shunting presented as early filling of the draining veins. Gradient echo hemosiderin sensitive sequences, such as T2* sequences, may help detect previous bleeding in intrasynovial venous malformations. Restricted diffusion is not present in venous malformations, hence diffusion-weighted imaging is not included in our routine protocol.

In this study, the main differential diagnoses offered for venous malformations were juvenile idiopathic arthritis, pigmented villonodular synovitis and hemangioma. Although mild synovial effusions may be present in venous malformations, the synovium itself does not enhance after gadolinium as it does in inflammatory synovitis, such as rheumatoid arthritis. In pigmented villonodular synovitis, the synovial thickening has different morphology and a more heterogeneous T2 signal than in venous malformations, although hemosiderin deposits are possible in both entities.

Hemangioma is a commonly used misnomer for venous malformations in many vascular anomaly publications [15, 16] and was also presented as an initial diagnosis among some of our cases. Also, intra-articular venous malformations of the knee may be misleadingly designated as synovial hemangiomas in the literature [17–20]. On MRI, hemangiomas are solid lesions with uniformly high signal intensity in T2-weighted sequences, prominent feeding arteries and draining veins, and intense and homogeneous enhancement after gadolinium. These features permit ready differentiation from venous malformations on imaging. Furthermore, most hemangiomas are of the infantile type, following a course of initial growth during the first few months of life followed by spontaneous involution. In contrast, venous malformations grow commensurately with the child [7] and may not become symptomatic until later childhood. Moreover, hemangiomas are not practically encountered in the knee joint in our experience.

Only a few studies of intra-articular venous malformations have distinguished between intracapsular but extrasynovial and intrasynovial lesions [4, 5, 21], although this is a clear determinant of the risk of chondral damage. In association with an intra-articular venous malformation, mild synovial effusion, hemosiderin deposits and thinning of cartilages are signs of recurrent bleeding and should raise a strong suspicion of an intrasynovial lesion. An important learning point is that synovial involvement may not always be evident on MRI, especially in the absence of a synovial effusion (Fig. 2). As shown by our data, it should be suspected in suprapatellar lesions with close proximity to the synovium of the suprapatellar recess and in diffuse lesions invading the intercondylar space near cruciate ligaments (Figs. 4 and 5). In our series, two patients with lesions of the suprapatellar fat pad had involvement of the suprapatellar recess not visible on MRI but detected at surgical treatment. It is important to note that the suprapatellar recess, also referred to as the suprapatellar synovial bursa, is continuous with the joint cavity and lies immediately posterior to the suprapatellar fat pad. In intra-articular venous malformations of the knee, clinicians should be made aware of this potential limitation of MRI. Diagnostic arthroscopy is one option for further investigating the synovium in unclear cases.

## Conclusion

Intra-articular venous malformations of the knee are a rare but important condition in the pediatric population. The clinical picture in children may be nonspecific and the symptoms and signs display overlap with other conditions. Although MRI usually permits the diagnosis, familiarity with vascular malformations is important for optimal imaging and accurate interpretation. Involvement of the intrasynovial cavity is a risk for hemarthrosis and progressive chondropathy but possibly underestimated by magnetic resonance images.
